# Investigating pigeon circovirus infection in a pigeon farm: molecular detection, phylogenetic analysis and complete genome analysis

**DOI:** 10.1186/s12864-024-10303-4

**Published:** 2024-04-16

**Authors:** Xiaobo Li, Shujing Wang, Wei Li, Shasha Wang, Xiao Qin, Ji Wang, Rui Fu

**Affiliations:** 1 Institute of Laboratory Animal Resources, National Institutes for Food and Drug Control, Beijing, 102629 People’s Republic of China; 2 National Rodent Laboratory Animal Resources Center, National Institutes for Food and Drug Control, Beijing, 102629 People’s Republic of China; 3 National Laboratory Animal Quality Testing Center, National Institutes for Food and Drug Control, Beijing, 102629 People’s Republic of China

**Keywords:** Pigeon circovirus, Genetic diversity, Phylogenetic analysis, Recombination analysis

## Abstract

**Background:**

Pigeon circovirus infections in pigeons (*Columba livia domestica*) have been reported worldwide. Pigeons should be PiCV-free when utilized as qualified experimental animals. However, pigeons can be freely purchased as experimental animals without any clear guidelines to follow. Herein, we investigated the status quo of PiCV infections on a pigeon farm in Beijing, China, which provides pigeons for experimental use.

**Results:**

PiCV infection was verified in at least three types of tissues in all forty pigeons tested. A total of 29 full-length genomes were obtained and deposited in GenBank. The whole genome sequence comparison among the 29 identified PiCV strains revealed nucleotide homologies of 85.8–100%, and these sequences exhibited nucleotide homologies of 82.7–98.9% as compared with those of the reference sequences. The *cap* gene displayed genetic diversity, with a wide range of amino acid homologies ranging from 64.5% to 100%. Phylogenetic analysis of the 29 full-genome sequences revealed that the PiCV strains in this study could be further divided into four clades: A (17.2%), B (10.4%), C (37.9%) and D (34.5%). Thirteen recombination events were also detected in 18 out of the 29 PiCV genomes obtained in this study. Phylogenetic research using the *rep* and *cap* genes verified the recombination events, which occurred between clades A/F, A/B, C/D, and B/D among the 18 PiCV strains studied.

**Conclusions:**

In conclusion, PiCV infection, which is highly genetically varied, is extremely widespread on pigeon farms in Beijing. These findings indicate that if pigeons are to be used as experimental animals, it is necessary to evaluate the impact of PiCV infection on the results.

**Supplementary Information:**

The online version contains supplementary material available at 10.1186/s12864-024-10303-4.

## Background

The pigeon (*Columba livia domestica*), as a commonly used experimental animal in scientific research, has several advantages such as quick reproduction, a short incubation cycle, strong flight ability, low breeding cost, easy in vitro incubation, and easy acquisition of embryonic cells. In life science research, pigeons can be used for research on disease models [1], etiology [2, 3], and goal-oriented neural pathways [4], indicating enormous potential value in vaccine research and development, drug clinical trials, and disease research [5]. The precision and dependability of the experimental results are directly proportional to the quality of the pigeon data used. There are guidelines for the domestic supervision of experimental pigeons. As a crucial component of quality control strategies, pathogens, especially the common pigeon circovirus (PiCV), must be controlled. In this study, we looked at the current state of PiCV infections on a pigeon farm in Beijing, China, where pigeons are raised for research purposes.

Pigeon circovirus (PiCV) is a member of the *Circovirus* genus in the *Circoviridae* family, which includes small, non-enveloped viruses with a diameter of around 20 nm. The PiCV genome is a single-stranded circular DNA sequence of 1.7–2.5 kilobases (kb) [6]. All circoviruses have two major open reading frames (ORFs). The largest ORF, V1, which is located on the virion sense strand, encodes a protein responsible for viral replication (Rep protein), and the second largest ORF, C1, which is located on the complementary sense strand, encodes the viral capsid protein (Cap protein) [7–10].

PiCV can be transferred horizontally through feces as well as vertically [11], and it is thought to be a possible cause of young pigeon sickness syndrome (YPDS). The diseased pigeons are mostly young pigeons aged 2–12 months, with typical symptoms, including drowsiness, weight loss, anorexia, work reflux, excessive drinking, and diarrhea. The virus can also harm pigeons' immune systems, making them more vulnerable to reinfection by numerous conditional pathogens, causing immunological organ deterioration, and producing pathological alterations such as inflammation in the trachea, liver, lungs, and intestines [12, 13]. Circovirus infection in pigeons was first diagnosed in 1993 in the USA [14]. To date, except for porcine circovirus, which can reproduce in cells such as PK15, all other circoviruses have not been successfully cultivated [15, 16]. Moreover, PiCV detection relies mainly on PCR methods [17–22]. Cases of PiCV infections in pigeons have been reported in various countries and regions, such as Northern Ireland [10], Germany [8, 23], Italy [24], France [25], the Czech Republic [26], Belgium [11, 27], Poland [28, 29], Slovenia [30], Hungary [6], the United Arab Emirates [31], Iran [32], China [22, 33, 34], Japan [35], the USA [14, 19], Brazil [12] and Australia [36]. Because of the high incidence of PiCV in both domesticated and feral urban pigeon populations, PiCV infections are among the most significant health-related difficulties faced during the breeding of these birds [28, 30, 37]. In China, the first PiCV infection was detected in meat pigeons in Zhejiang Province in 2009 [38]. In recent years, several studies have proven that PiCV is prevalent among pigeons in China [22, 33, 37, 39]. Pigeons are widely employed as food, racing, carrier, and experimental animals. Considering the impact of PiCV on experimental results, especially those related to the immune system [40, 41], pigeons should be free of PiCV infection. However, pigeons can be purchased as experimental animals without certificateing that they are PiCV free. The current study sought to explore the state of PiCV infections and analyze the viruses' complete genome sequences on a pigeon farm in Beijing, China.

## Methods

### Sample collection and PiCV detection

The study was carried out according to the ethical review guidelines for animal welfare (GB/T 35892) and approved by the Ethics Committee for Laboratory Animal Welfare of the National Institutes for Food and Drug Control [Approval No. NIFDC(FU)2021(A)007]. Forty carrier pigeons, numbered #1-#40 and aged 3 to 4 months and with a healthy appearance, were obtained from a breeding farm in Fangshan District, Beijing, China. There were approximately 200 pigeons in the pigeon farm. The 40 pigeons were taken from different breeding units, which can represent the overall animal condition of the pigeon farm. After euthanasia by carbon dioxide suffocation, the liver, spleen, lung, bursa of Fabricius, and cecum were aseptically collected. Approximately 1 g of each sample was macerated with 900 μl of sterilized phosphate-buffered saline (PBS, pH 7.2), fully homogenized, and centrifuged at 12,000 × g for 5 min. A total of 0.2 ml of supernatant was used for DNA extraction using an IndiSpin QIAcube HT Pathogen Kit (QIAGEN, Leipzig, Germany) according to the manufacturer's instructions, and the extracted DNA was kept frozen at -20 °C before use. PiCV nucleic acid was detected using PiCV Taqman real-time fluorescent PCR [42]. Specific primers and probe for PiCV were developed from the Rep protein region of the viral genome (GenBank accession no. NC_002361): the upstream primer 5′-GGGCCTTTGTGGAGTTCAC-3′ (Location: 807–825 nt); the downstream primer 5′-CACCTTTCCTCGCTGTACCA-3′ (Location: 888–869 nt); probe 5′-(FAM)ACCAGCAACAGTCCCC(NFQ)-3′ (Location: 845–860 nt). A reaction mixture was prepared to contain (per vial) 2 μl of sample DNA, 0.5 μM of each primer and 0.25 μM of the probe, 10 μl of 2 × TaqMan Universal PCR Master Mix (ThermoFisher Scientific, Carlsbad, California, USA), and the volume was adjusted to 20 μl with nuclease-free water. The cycling parameters for PCR included 2 min at 50 °C, 10 min at 95 °C for enzyme activation, followed by 40 cycles for amplification with 95℃ for 15 s and 60℃ for 1 min. Fluorescence signals were collected at the end of each amplification step. The reaction was carried out using the ABI7500 Fast Real-Time PCR System and software (Applied Biosystems Inc., Foster City, CA, USA). A piece of pigeon liver tissue PiCV negative was used as a negative control, and sterilized double distilled water (ddH_2_O) was used as a blank control. A sample was judged as PiCV positive if there was an obvious amplification curve with a Ct value ≤ 40; otherwise, it was considered negative. PiCV-positive samples (mainly from the bursa of Fabricius) were subjected to genome sequencing. Simultaneously, the pigeon samples were screened for Newcastle disease virus, avian influenza virus, pigeon pox virus, fowl adenovirus, Chlamydia, *Salmonella* sp., and *Pasteurella multocida* (the methods used are shown in Supplementary File 1, Table S1).

### Full-length genome amplification and sequencing

The full PiCV genome of all the positive samples was amplified via PCR using the following primers [29]: PiCV-AV-F (5′-TCGCGCGAGASTTCAGTGARAT-3′) and PiCV-AV-R (5′-CYTCSGYCATTGCTCTTCCGGCTTTC-3′). These primers can amplify the complete genomes of all the PiCV variants. PrimeSTAR GXL DNA Polymerase (Takara, Japan) was used for the amplification using the following thermal cycling conditions: 30 cycles of 98 °C (10 s), 60 °C (15 s) and 68 °C (2 min). The contents of the reaction mixture were as follows: 10 μl of 5 × PrimeSTAR GXL buffer, 200 nM of each primer, 4 μl of dNTP mixture (2.5mM each), 1.25U of PrimeSTAR GXL DNA Polymerase, 2 μl DNA. The final reaction volume was made up to 50 μl with nuclease-free water. After agarose gel electrophoresis, the PCR amplicons were excised and purified with a SanPrep Column DNA Gel Extraction Kit (Sangon Biotech, Shanghai, China) and ligated to the pESI-T vector using Hieff Clone TM Zero TOPO-TACloning Kit (Yeasen Biotech, Shanghai, China) after adding A tail. The resulting plasmids obtained for each of the PiCV genome isolates from a single transformed *E. coli* colony were Sanger sequenced at ABI3730 using pESI-T-specific sequencing primers (M13F: TGTAAAACGACGGCCAGT /M13R: CAGGAAACAGCTATGACC) and internal sequencing primers (Supplementary File 2, Table S2). The contigs were assembled using the SeqMan program of DNASTAR software (version 7.1.0).

### Genome characterization and phylogenetic analysis of the PiCV genome

A total of twenty-nine full genome sequences were obtained in this study and deposited in GenBank (ON598384-ON598388, OR843255-OR843278). The ORFs in the sequence were predicted using the web program NCBI ORF Finder (National Center for Biotechnology Information, https://www.ncbi.nlm.nih.gov/orffinder/). The sequences obtained in the present study were compared with the PiCV reference dataset available in the National Center for Biotechnology Information (NCBI) nucleotide database (Supplementary File 3, Table S3). Multiple sequence alignments were carried out by the ClustalW algorithm using MEGA 5.2 software, and the homology among nucleotide and amino acid sequences was determined by using BioEdit (v.7.0.9.0) software. The entire genome sequence of PiCV obtained in this study was used for phylogenetic analysis. PiCV sequence data from different geographical locations within China (*n* = 144) and the rest of the world (*n* = 72) were retrieved from the NCBI nucleotide database as reference sequences (Supplementary File 3, Table S3). The phylogenetic tree was constructed in MEGA5.2 using the neighbor-joining method (bootstrapping with 1000 replications) and the Kimura 2-parameter model. The evolutionary tree was rooted with a beak and feather disease virus (BFDV) genome (GenBank accession number AF071878) and generated and annotated with Interactive Tree Of Life (iTOL) software (http://itol.embl.de/). Recombination analysis indicated that the recombination breakpoint hot spots were located within both the intergenic region between the *rep* and *cap* stop codons and near the virion strand of origin during replication. Therefore, phylogenetic trees based on the *Cap* and *Rep* genes were also inferred using the same parameters as above.

### Recombination analysis

To analyze the potential recombinant evidence, an integrated software package for the recombination detection program RDP4 [43] was used to detect potential recombinant strains, parental strains, and possible recombination breakpoints. The complete genome sequences were aligned with those used by Wang et al. [39]. The final dataset comprising 209 sequences was screened. Seven methods (RDP, GENECONV, BootScan, MaxChi, Chimera, SiScan, and 3Seq) were used. Recombination events were identified by at least three out of the seven detection methods, and a *P*-value < 0.05 was considered to indicate plausible recombinant events. Recombination breakpoint distribution plots were analyzed and automatically generated by the software RDP4.

## Results

### Real-time fluorescent PCR testing of PiCV in pigeon samples

**Fig. 1:**
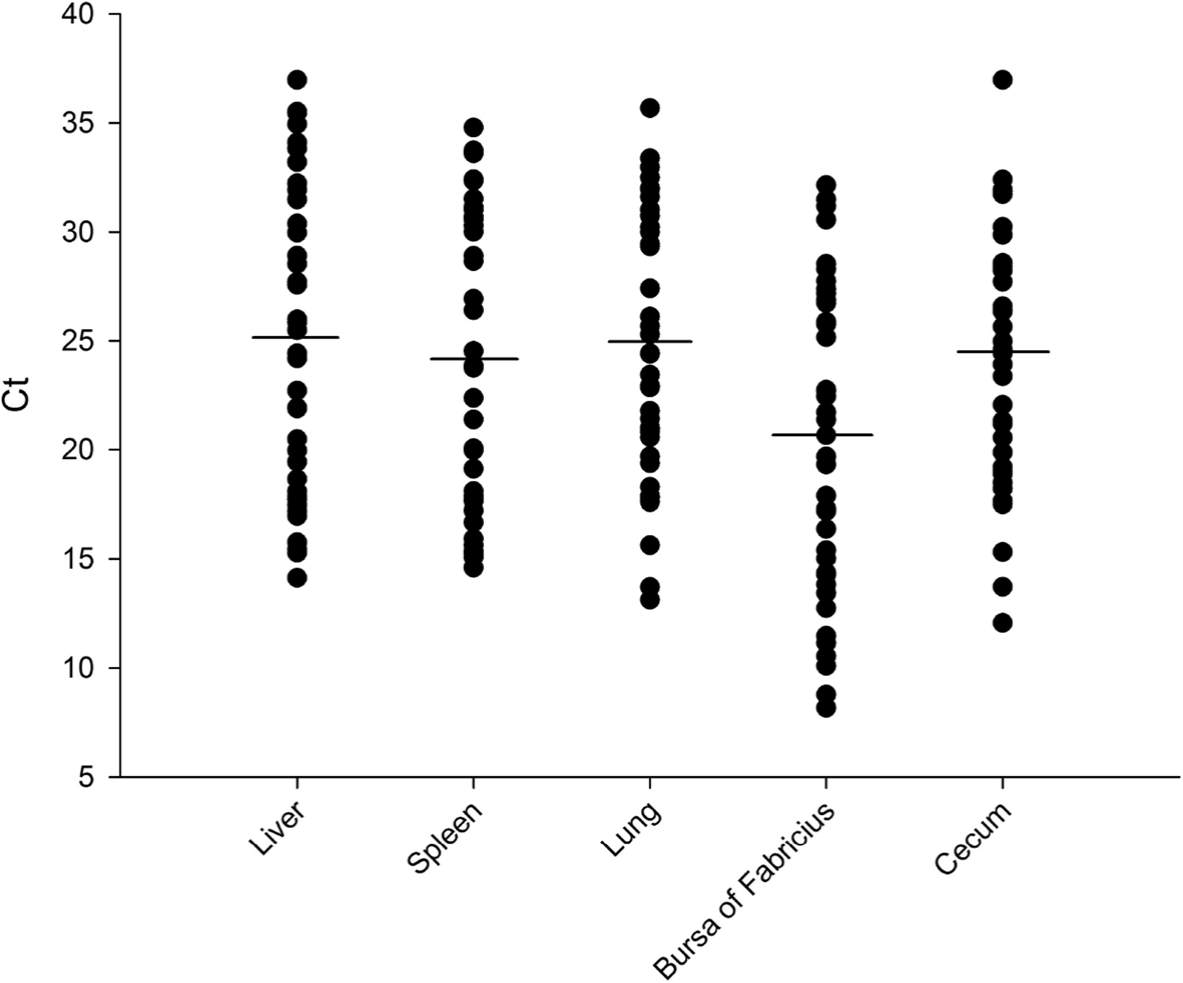
Fluorescent PCR Ct values of different tissues of 40 pigeons

The genomic DNA of 196 samples was amplified using real-time fluorescent PCR for PiCV. The samples included forty liver, spleen, lung, and bursa of Fabricius samples and 36 cecum samples. The percentage of positive PiCV results was 97.5% (39/40) for liver tissue, 100% (40/40) for spleen tissue, 100% (40/40) for lung tissue, 100% (40/40) for bursa of Fabricius, and 97.2% (35/36) for cecum tissue. The mean Ct value for the bursa of Fabricius (Ct value: 20.4) was lower than that for the other tissues (liver 24.9, spleen 23.7, lung 24.7, and cecum 24.0) (Fig. 1). Then, the bursa of Fabricius samples tested PiCV-positive were subjected to genome sequencing. Moreover, the pigeon samples were screened for Newcastle disease virus, avian influenza virus, pigeon pox virus, fowl adenovirus, Chlamydia, *Salmonella* sp., and *Pasteurella multocida,* and the results were all negative.

### Sequencing of PiCV-positive samples and genome characterization

Using the primer pairs (PiCV-AV-Fand PiCV-AV-R), we collectively recovered 29 complete PiCV genomes from the bursa of Fabricius (*n* = 28) and lung samples (*n* = 1) (Table 1). The assembled circular whole-genome sequences were detected as seven sizes ranging from 2032 to 2042 nt. Among these variable genome lengths, the most common genome sizes are 2037 (*n* = 12) and 2041 (*n* = 8) nucleotides (Table 1). The 5′ intergenic region, found between the start sites of the two major ORFs, contains the potential stem-loop at which rolling circle replication (RCR) of the virus DNA strand is postulated to begin. There is a stem ring structure between the beginning of ORF V1 and ORF C1, which has a conserved 9-base sequence TAGTATTAC. Many insertions, deletions, and substitutions have been discovered during the analysis of the PiCV genome nucleotide sequence, but no mutations were found in the conserved 9-base sequence, which may be related to the initiation of RCR. There is also an inverted repeat near the stem ring (CACGGAGCCAC*ATCGC and GCGATA*TGGCTCCGTG, where the base marked by "*" is not completely paired). This sequence may constitute the binding site for the PiCV replicase. In addition, there is a forward repeat sequence GGAGCC near the stem ring. The full genome sequence homology among the 29 PiCV isolates is 85.8–100%. The 29 PiCV isolates have nucleotide homology greater than 84.2% with the Chinese reference strain and greater than 82.7% with reference strains from other countries (Table 2).

The 29 identified *cap* genes have three sizes, 813 nt (*n* = 3), 819 nt (*n* = 3), and 822 nt (*n* = 23), with deduced amino acid sequence lengths of 270, 272, and 273 aa, respectively (Table 1). Sequence comparison of the 29 *cap* genes revealed nucleotide homologies of 77.0–100% and deduced amino acid homologies of 76.6–100%. These sequences exhibit greater similarities with PiCV reference strains from China (72.8–99.5% nucleotide identity, 72.6–100% amino acid identity) than with PiCV reference strains from other countries (70.7–99.2% nucleotide identity, 64.5–100% amino acid identity) (Table 2). The start codons encoding 29 isolated PiCV Cap proteins are all "ATG", although other starting codons have been reported [22, 39]. The amino acid sequences of 29 identified Cap proteins and the reference strains were aligned to investigate variations in the deduced amino acid sequences. The results identified 10 large deletion sites (relative to the consensus sequence) among the Cap proteins, including residues 7, 28, 29, 30, 37, 53, 130, 182, 266, and 267, as well as four common replacement regions.

**Table 1:** Information on the pigeon circovirus (PiCV) strains in this study

No	Strain name	Source sample	Genome length (nt)	Rep (aa)	*rep* gene length (nt)	Cap (aa)	*cap* gene length (nt)	Accession number
1	Fa28/Beijing/2021	bursa of Fabricius	2038 (41–994)^a^ (1983–1165) ^b^	317^c^	954	272	819	ON598384
2	Fa29/Beijing/2021	bursa of Fabricius	2037 (41–994) (1987–1166)	317	954	273	822	ON598385
3	Fa33/Beijing/2021	bursa of Fabricius	2037 (48–995) (1987–1166)	315	948	273	822	ON598386
4	Fa39/Beijing/2021	bursa of Fabricius	2035 (41–991) (1980–1162)	316	951	272	819	ON598387
5	Lu27/Beijing/2021	lung	2038 (41–994) (1983–1165)	317	954	272	819	ON598388
6	Fa1/Beijing/2021	bursa of Fabricius	2032 (48–995) (1978–1166)	315	948	270	813	OR843255
7	Fa2/Beijing/2021	bursa of Fabricius	2032 (48–995) (1978–1166)	315	948	270	813	OR843256
8	Fa3/Beijing/2021	bursa of Fabricius	2041 (41–994) (1986–1165)	317	954	273	822	OR843257
9	Fa4/Beijing/2021	bursa of Fabricius	2041 (41–994) (1986–1165)	317	954	273	822	OR843258
10	Fa5/Beijing/2021	bursa of Fabricius	2041 (41–994) (1986–1165)	317	954	273	822	OR843259
11	Fa6/Beijing/2021	bursa of Fabricius	2041 (41–994) (1986–1165)	317	954	273	822	OR843260
12	Fa8/Beijing/2021	bursa of Fabricius	2041 (41–994) (1986–1165)	317	954	273	822	OR843261
13	Fa11/Beijing/2021	bursa of Fabricius	2041 (41–994) (1986–1165)	317	954	273	822	OR843262
14	Fa15/Beijing/2021	bursa of Fabricius	2037 (41–994) (1987–1166)	317	954	273	822	OR843263
15	Fa17/Beijing/2021	bursa of Fabricius	2037 (41–994) (1987–1166)	317	954	273	822	OR843264
16	Fa19/Beijing/2021	bursa of Fabricius	2037 (41–994) (1987–1166)	317	954	273	822	OR843265
17	Fa21/Beijing/2021	bursa of Fabricius	2042 (48–995) (1987–1166)	315	948	273	822	OR843266
18	Fa23/Beijing/2021	bursa of Fabricius	2037 (41–994) (1987–1166)	317	954	273	822	OR843267
19	Fa25/Beijing/2021	bursa of Fabricius	2037 (41–994) (1987–1166)	317	954	273	822	OR843268
20	Fa26/Beijing/2021	bursa of Fabricius	2037 (41–994) (1987–1166)	317	954	273	822	OR843269
21	Fa27/Beijing/2021	bursa of Fabricius	2037 (41–994) (1987–1166)	317	954	273	822	OR843270
22	Fa30/Beijing/2021	bursa of Fabricius	2037 (48–995) (1987–1166)	315	948	273	822	OR843271
23	Fa31/Beijing/2021	bursa of Fabricius	2041 (48–995) (1987–1166)	315	948	273	822	OR843272
24	Fa32/Beijing/2021	bursa of Fabricius	2037 (41–994) (1987–1166)	317	954	273	822	OR843273
25	Fa35/Beijing/2021	bursa of Fabricius	2041 (48–995) (1987–1166)	315	948	273	822	OR843274
26	Fa36/Beijing/2021	bursa of Fabricius	2037 (41–994) (1987–1166)	317	954	273	822	OR843275
27	Fa37/Beijing/2021	bursa of Fabricius	2042 (48–995) (1987–1166)	315	948	273	822	OR843276
28	Fa38/Beijing/2021	bursa of Fabricius	2032 (48–995) (1978–1166)	315	948	270	813	OR843277
29	Fa40/Beijing/2021	bursa of Fabricius	2036 (41–994) (1986–1165)	317	954	273	822	OR843278

^a^ nt position at which the ORF of Rep starts and stops;

^b^ nt position at which the ORF of Cap starts and stops

^c^ Number of amino acids encoded by the ORF. All sequence information can be found in the databases at the National Center for Biotechnical Information (NCBI) (http://ncbi.nlm.nih.gov/) by accession number. The start codons encoding 29 isolated PiCV Cap proteins were all "ATG"

**Table 2:** Sequence analysis of the genome sequences of pigeon circovirus (PiCV) strains

Selected strains	Genome sequence	*cap* gene sequence		*rep* gene sequence	
	**Nucleotide**	**Nucleotide**	**Amino acids**	**Nucleotide**	**Amino acids**
Identity of the PiCV strains identified in our study	85.8–100%	77.0–100%	76.6–100%	90.9–100%	91.1–100%
Compared with Chinese PiCV reference strains	84.2–98.9%	72.8–99.5%	72.6–100%	89.2–99.0%	88.6–99.3%
Compared with PiCV reference strains from other countries	82.7–97.9%	70.7–99.2%	64.5–100%	89.4–97.5%	90.2–99.0%

Tables 1 and S2 show the GenBank accession numbers of the PiCV strains from this study and PiCV reference strains from China and other countries, respectively

There are two main sizes of the *rep* gene: 948 nt (*n* = 9) and 954 nt (*n* = 19), except for ON598387 (951 nt), which encodes Rep proteins with 315, 317 and 316 residues (Table 1). The difference in size is due to a 2-amino acid deletion at positions 2 and 3. Sequence alignment revealed that the *rep* gene (90.9–100% nucleotide identity, 91.1–100% amino acid identity among 29 *rep* genes) is more conserved than the *cap* gene. Aligning the amino acid sequences of 29 *rep* genes and the reference sequences, we found that in all 29 identified Rep proteins, seven amino acid motifs putatively associated with RCR are completely conserved: FTLNNP (positions 41–46), TPHLQG (positions 76–81), YCSK (positions 116–119), G-GKS (positions 195–199), WWDGY (positions 217–221), DDFYGWLP (positions 230–237) and DRYP (positions 246–249).

### Phylogenetic and recombination analysis of genome sequences

**Fig. 2:**
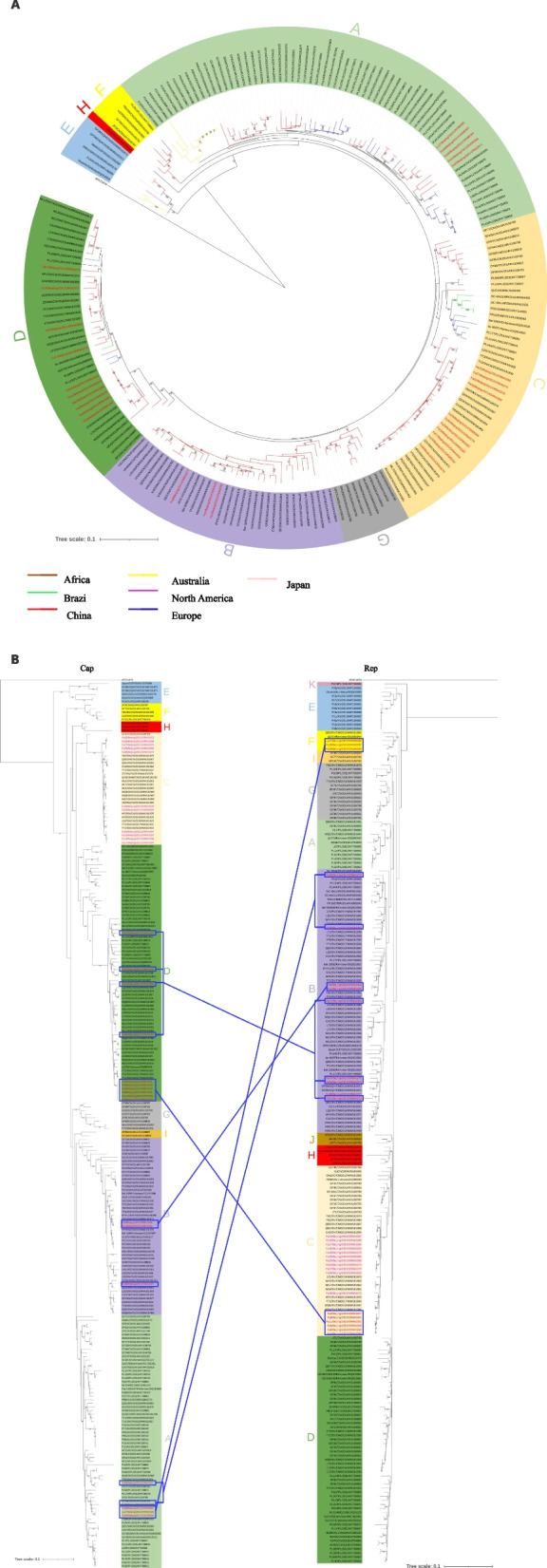
Phylogenetic tree based on the nucleotide sequences of the PiCV full genome (**A**) and the *cap* and *rep* genes (**B**). The tree was constructed using the neighbor-joining method, with bootstrap values calculated for 1,000 replicates, and was rooted with the BFDV genome (AF071878). Bootstrap values greater than 50 are shown. The labels at the branch tips refer to the strain name and GenBank accession number, with different background colors representing different clades. The red taxa highlight the 29 PiCV strains isolated in this study. The branches are labeled with different colors to represent different countries or regions

A phylogenetic analysis of 180 PiCV full-genome sequences from GenBank (Supplementary File 3, Table S3) and 29 PiCV strains obtained in our study was performed to investigate genetic relationships. The reference dataset included 120 full-genome sequences from China, 39 reference sequences from Europe, 12 reference sequences from Australia, 5 reference sequences from Brazil, 3 reference sequences from the United States, and 1 full-genome sequence from Japan. The corresponding phylogenetic tree was constructed using the sequence of Beak and Feather Disease Virus, a member of the family Circoviridae, as the outgroup. A total of 209 PiCV strains were divided into 8 clades (A-H) (Fig. 2 A). The 29 full genome sequences reported in this study were clustered into four clades: A (17.2%), B (10.4%), C (37.9%), and D (34.5%), although all the sequences were obtained from the same pigeon farm. In clade A, OR843271, ON598386, and 10 PiCV strains from Europe were in the same branch, and ON598384, ON598387, and ON598388 clustered with 3 PiCV strains from China and Europe. Clade B consisted of OR843255, OR843256, OR843277, 29 PiCV strains from China, and 2 strains from Europe. In clade C, 11 PiCV strains identified in this study clustered with 16 other PiCV strains from China. In clade D, 10 PiCV strains obtained in this study clustered with other PiCV strains from China except OR843272, which was closest with KF738849 from Europe (Fig. 2A).

**Fig. 3:**
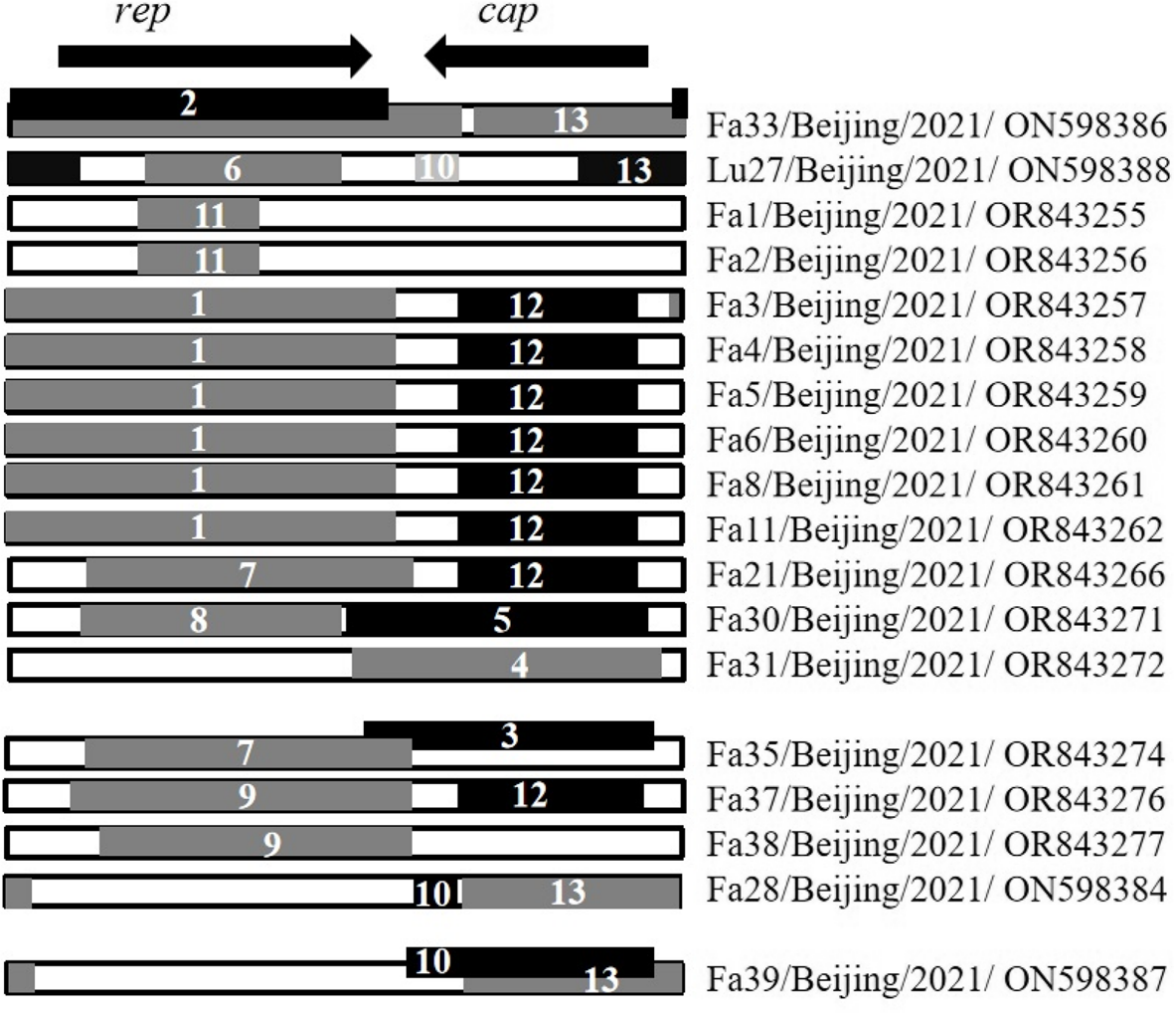
Map of recombination events detected in the Pigeon Circovirus (PiCV) strains obtained in this study. The recombination event numbers correspond to Table S4

**Fig. 4:**
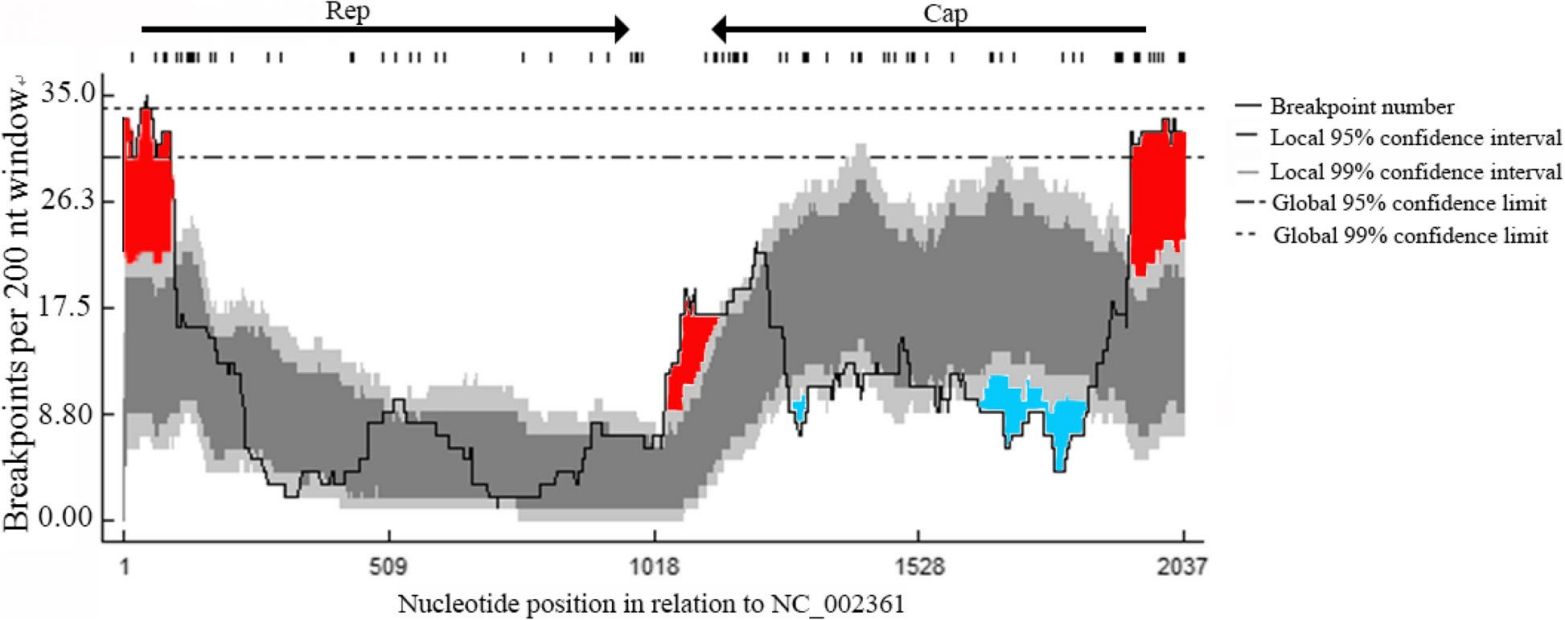
Recombination breakpoint distribution plots for 29 PiCV full-genome sequences identified in this study and 180 PiCV full-genome sequences available in GenBank. The red and blue areas of the plots indicate recombination breakpoint hot spots and cold spots, respectively. The dark and light gray areas represent the 95% and 99% confidence intervals, respectively, of the expected degrees of breakpoint clustering under random recombination

The 29 PiCV full-genome sequences obtained in this study and 180 PiCV reference sequences in the NCBI database (Supplementary File 3, Table S3) were analyzed using RDP software after alignment via BioEdit software. A total of thirteen recombination events related to the 18 PiCV strains were detected in the present study (Supplementary File 4, Table S4). In some cases, evidence of two or more independent events was detected within individual genomes; for example, two independent recombination events (Events 7 and 12) were detected within Fa21/Beijing/2021/OR843266 (Fig. 3). Additionally, 11 PiCV genomes (Fa15/Beijing/2021/OR843263, Fa17/Beijing/2021/OR843264, Fa19/Beijing/2021/OR843265, Fa23/Beijing/2021/OR843267, Fa25/Beijing/2021/OR843268, Fa26/Beijing/2021/OR843269, Fa27/Beijing/2021/OR843270, Fa29/Beijing/2021/ON598385, Fa32/Beijing/2021/ON598385, Fa36/Beijing/2021/OR843275 and Fa40/Beijing/2021/OR843278) had no evidence of recombination. The potential parents came from China, including northern provinces such as Shaanxi; southern regions such as Zhejiang and Jiangsu; and Qinghai in Northwest China, as well as from Europe, Oceania, and South America. The results revealed the different evolutionary origins of PiCV from the perspective of genetic recombination. Some PiCV strains isolated in the present study were detected as potential parents at various events (Supplementary File 4, Table S4), indicating the occurrence of viral recombination in the local pigeon population. We also analyzed the possible breakpoints for recombination. The recombination breakpoint hot spots were located near the virion strand origin of replication, and the intergenic region between the *rep* and *cap* stop codons (Fig. 4). The recombinations were subsequently confirmed via phylogenetic analysis of the *rep* and *cap* genes. The 11 PiCV genomes that had no evidence of recombination were clustered into the same clade (clade C) in both the *rep* and *cap* gene phylogenetic trees. The other 18 PiCV strains with detected recombination signals were clustered into different clades according to *rep* and *cap* gene phylogenetic analysis, except for Fa1/Beijing/2021, Fa2/Beijing/2021, and Fa38/Beijing/2021 (Fig. 2B). Briefly, six PiCV strains were clustered into clade D in the *cap* gene phylogenetic tree and clade C in the *rep* gene. The four PiCV strains were clustered into clades D and B in the *cap* and *rep* gene phylogenetic trees, respectively. The *cap* gene phylogenetic study placed five PiCV strains in clade A, while the *rep* gene phylogenetic tree placed them in clade B or F (Fig. 2B).

## Discussion

PiCV infection is a big barrier preventing pigeons from being one of the best experimental animal models in Beijing, China. First, all 40 pigeons were from the same breeding farm in Beijing, with a PiCV positivity rate of 100%, but other pathogens, such as Newcastle disease virus, avian influenza virus, pigeon pox virus, fowl adenovirus, Chlamydia, *Salmonella* sp., and *Pasteurella multocida*, were all negative (data not shown). Second, PiCV infection affects several tissues, including the liver, spleen, lung, bursa of Fabricius, and cecum, suggesting that tissue-targeted functional studies may be relevant [7, 22, 30, 33, 44]. Third, none of these 40 pigeons exhibited obvious clinical signs, indicating that PiCV is a latent infection in the pigeon population [12, 30], which may result in apparently healthy PiCV-carrying pigeons being treated as qualified experimental animal models.

The full-length genome analysis revealed a variety of PiCV traits, including genetic diversity, insertions, deletions, substitutions, and recombination. The assembled circular whole-genome sequences were detected as seven sizes ranging from 2032 to 2042 nt in length, consistent with the findings of previous studies [12, 22, 36, 39]. Despite several insertions, deletions, and substitutions detected during the examination of the PiCV genome nucleotide sequence, no mutation was observed in the conserved 9-base sequence of the TAGTATTAC motif situated at the stem ring structure, which may be associated with the beginning of RCR [8, 10]. The 29 PiCV strains obtained in this study have ten single amino acid deletions in Cap. Substitution of amino acids is relatively common, with four frequent substitution regions. The greater diversity of the Cap protein is because the Cap protein, as the protein shell of the virus, is exposed to the host immune cells, and positive selection occurs during this process. Some mutations may affect the structure of the Cap protein, resulting in enhanced virus binding to receptors on target cells, boosting the virus's potential to infect cells..Furthermore, these changes may shield viruses from neutralization by antibodies [39, 44]. Previous studies have shown that recombinant Cap protein can induce humoral and cellular immunity in pigeons and can be considered a potential antigen candidate for subunit vaccines against PiCV infections [45, 46]. According to the reasoning provided above, mutations in the Cap protein may impact the vaccine's immunological effectiveness. Due to the lack of suitable culture conditions in vitro for PiCV growth [15, 16], more studies on the pathogenic mechanism of PiCV are needed. The pathogenesis of PiCV is currently unclear, and further research is needed.

Phylogenetic analysis of the full-length genome revealed that a total of 209 examined PiCV strains could be grouped into 8 clades (A-H), consistent with previously reported findings [35, 39]. With the increase in the number of sequences in public databases, additional phylogenetic clades are expected to emerge. A previous study showed that PiCV strains isolated from the same club belonged to different clades and shared low sequence identity [39]. The 29 PiCV strains obtained in this study were divided into four clades, although they were obtained from the same pigeon farm. These data suggest that the infection and evolution of PiCV on pigeon farms have different evolutionary origins, which may result from the frequent trade of pigeons and warns for the need of more oversight. The results also indicate that, as with BFDV, long-distance geographical movements are likely to have occurred relatively frequently during the evolution of PiCV, and geographical and temporal factors have almost no effect on the genome diversity of PiCV [22, 29]. Interestingly, two isolates, Lu27/Beijing/2021/ON598388 (clade A, from the lung) and Fa27/Beijing/2021/OR843270 (clade C, from the Bursa of Fabricius), with lower identity (81.3% nucleotide identity and 87.5% amino acid identity in the *cap* gene), were detected from the same pigeon. These findings are similar to prior studies [39], indicating that PiCV was transmitted horizontally on the same pigeon farm.

Additionally, viral recombination has been shown to play a crucial role in the evolution of several ssDNA viruses [47]. A total of thirteen events related to 18 out of the 29 identified PiCV strains were detected via recombination analysis. Most of their potential parents came from China, including northern provinces such as Shaanxi; southern regions such as Zhejiang and Jiangsu; and Qinghai in Northwest China, as well as from Europe, Oceania, and South America. The results revealed the different evolutionary origins of PiCV from the perspective of genetic recombination. Some PiCV strains obtained in the current investigation were identified as prospective parents at various events, demonstrating the presence of viral recombination in the local pigeon population. We also analyzed the possible breakpoints for recombination. The results showed that the recombination breakpoint hot spots were located near the virion strand origin of replication and the intergenic region between the *rep* and *cap* stop codons, which were consistent with the results of Wang et al. [39]. As a result, a phylogenetic study of the *rep* and *cap* genes was conducted. We found that the 11 PiCV genomes that had no evidence of recombination were clustered into the same clade (clade C) in both the *rep* and *cap* gene phylogenetic trees, and the other 18 PiCV strains for which recombination signals were detected were clustered into different clades in the *rep* and *cap* gene phylogenetic analyses, except for three PiCV strains (Fa1/Beijing/2021, Fa2/Beijing/2021 and Fa38/Beijing/2021). The recombination breakpoints Fa1/Beijing/2021 and Fa2/Beijing/2021 were located inside the *cap* gene (318–528 nt). The exact breakpoint position of Fa38/Beijing/2021 was undetermined, and the recombinant sequence may have been misidentified (Supplementary File 4, Table S4), which explains why these three PiCV strains were located in the same clade based on the phylogenetic analysis of the *cap* and *rep* genes. These data clearly confirm the recombinant origins of various PiCV strains, which are most likely the product of genome recombination. This study and previous reports [39, 48] showed that there were two different PiCV strains in the same pigeon and that these two PiCV strains belonged to different branches. It is speculated that coinfection of different PiCV strains in the same pigeon is common and creates conditions conducive to virus recombination. The diversity of pandemic PiCV strains may make it difficult to protect pigeons against infection. It is vital to eradicate PiCV from the experimental pigeon population.

## Conclusion

In conclusion, our findings revealed that PiCV infection, which is highly genetically varied, is extremely widespread on a pigeon farm in Beijing. Furthermore, the identified PiCV strains exhibited high genetic diversity. Our data demonstrated that PiCV in Beijing pigeons underwent extensive recombination. PiCV's great genetic diversity may be due to the frequent trafficking of pigeons and a lack of control. Furthermore, the high intensity of recombination may suggest frequent coinfections of different PiCV strains. Overall, the diversity of pandemic PiCV strains may make pigeon infection prevention difficult. It is vital to eradicate PiCV from the experimental pigeon population.

### Supplementary Information

**Supplementary Material 1.**

## Data Availability

Sequence data that support the findings of this study have been deposited in  GenBank under accession numbers ON598384-ON598388 and OR843255-OR843278.

## Abbreviations

PiCV: Pigeon circovirus
YPDS: Young pigeon disease syndrome
BFDV: Beak and feather disease virus
ORF: Open reading frame
RCR: Rolling circle replication
RDP: Recombination Detection Program
PBS: Phosphate-buffered saline
iTOL: Interactive Tree Of Life
