# Supplementary material for: Investigating pigeon circovirus infection in a pigeon farm: molecular detection, phylogenetic analysis and complete genome analysis
Source: BMC Genomics. 2024 Apr 16;25:369. doi: 10.1186/s12864-024-10303-4 (PMC11020411; doi:10.1186/s12864-024-10303-4)
Supplement: Supplementary file 1 — Supplementary Material 1. [file 12864_2024_10303_MOESM1_ESM.docx]

**Supplementary Table 1**  Methods used for pigeon pathogens screening in this study

| Pathogens | Detection methods | References |
| --- | --- | --- |
| Newcastle disease virus | Forward primer: 5′-tatacgactcactatattgccagcacaaaaaggtctcc-3′  Reverse primer: 5′-tcatcaccacctatccattcatctt-3′  Probe: 5′VIC-cttcctaggcagagcat-TAMRA3′ | DB22/T 3031 One step fluorescent RT-PCR mehtod for simultaneous detection of the AIV and NDV |
| avian influenza virus, | Forward primer: 5′-tatacgactcactatatcttctaaccgaggtcgaaacg-3′  Reverse primer: 5′-ttcttccctgcaaagacatcttc-3′  Probe: 5′FAM-ccctcaaagccgagatcgcgc-TAMRA3′ | DB22/T 3031 One step fluorescent RT-PCR mehtod for simultaneous detection of the AIV and NDV |
| pigeonpox virus, | Forward primer: 5′- ctaataaactagaagcagaaataaatatgagatcaatcaaaag -3′  Reverse primer: 5′- tcagatagtctagattcacatgagatacaaaca -3′  Probe: 5′FAM- gtaagttcccatatcccgattatgt -NFQ3′ | Developed in our lab |
| fowl adenovirus | Forward primer: 5′-gccaccggaagctactttga-3′  Reverse primer: 5′-ttgtgatccatgggcatga-3′ | NY/T4027 Diagnostic methods for group I fowl adenovirus |
| Chlamydia | Forward primer1: 5′-atgaaaaaactcttgaaatcgg-3′  Reverse primer1: 5′-ttagaatctgaattgagcattcat-3′  Forward primer2: 5′-caggatactacggagattatgttt-3′  Reverse primer2: 5′-gattagattgagcgtattggaa-3′ | NY/T562 Diagnostic techniques for animal chamydiosis |
| *Salmonella sp.* | Isolating culture | GB/T14926.1 Laboratory animal- Method for examination of *Salmonella sp.* |
| *Pasteurella multocida* | Isolating culture | GB/T14926.5 Laboratory animal- Method for examination of *Pasteurella multocida* |

**Supplementary Table 2** Internal sequencing primers used for clone sequencing in this study

| No. | Strain name | Internal sequencing primers | Internal sequencing primer sequence(5'-3') |
| --- | --- | --- | --- |
| 1 | Fa28/Beijing/2021 | F1.3P/ F28.5p | >:F1.3P  TGGCGAGTCTGGCGGGTC  >:F1.5P  ACTAGCGGTATCTGATAGTCC  >:F3.5p  GACTTTTAGTACTGGTGCGTTC  >:F17.5p  GTTATCTCGGCCCCTATTGGTC  >:F28.5p  TGTGGCCGAATCCTTTCCAGGT  >:F30.5p  ACTAACGCAATCTGATAGTCCT  >:F31.5p  ATTTCCACCTTAACCAGCGGTA  >:F38.5p  CCTATTGGCCTCATATCCACCT  >:F21.5P  GACTTTCAATACTGGTGCGTTC |
| 2 | Fa29/Beijing/2021 | F1.3P/ F17.5p |  |
| 3 | Fa33/Beijing/2021 | F1.3P/ F28.5p |  |
| 4 | Fa39/Beijing/2021 | F1.3P/ F28.5p |  |
| 5 | Lu27/Beijing/2021 | F1.3P/ F28.5p |  |
| 6 | Fa1/Beijing/2021 | F1.3P/ F1.5P |  |
| 7 | Fa2/Beijing/2021 | F1.3P/ F1.5P |  |
| 8 | Fa3/Beijing/2021 | F1.3P/ F3.5p |  |
| 9 | Fa4/Beijing/2021 | F1.3P/ F3.5p |  |
| 10 | Fa5/Beijing/2021 | F1.3P/ F3.5p |  |
| 11 | Fa6/Beijing/2021 | F1.3P/ F3.5p |  |
| 12 | Fa8/Beijing/2021 | F1.3P/ F3.5p |  |
| 13 | Fa11/Beijing/2021 | F1.3P/ F3.5p |  |
| 14 | Fa15/Beijing/2021 | F1.3P/ F3.5p |  |
| 15 | Fa17/Beijing/2021 | F1.3P/ F17.5p |  |
| 16 | Fa19/Beijing/2021 | F1.3P/ F17.5p |  |
| 17 | Fa21/Beijing/2021 | F1.3P/ F21.5P |  |
| 18 | Fa23/Beijing/2021 | F1.3P/ F17.5p |  |
| 19 | Fa25/Beijing/2021 | F1.3P/ F17.5p |  |
| 20 | Fa26/Beijing/2021 | F1.3P/ F17.5p |  |
| 21 | Fa27/Beijing/2021 | F1.3P/ F17.5p |  |
| 22 | Fa30/Beijing/2021 | F1.3P/ F30.5p |  |
| 23 | Fa31/Beijing/2021 | F1.3P/ F31.5p |  |
| 24 | Fa32/Beijing/2021 | F1.3P/ F17.5p |  |
| 25 | Fa35/Beijing/2021 | F1.3P/ F3.5p |  |
| 26 | Fa36/Beijing/2021 | F1.3P/ F17.5p |  |
| 27 | Fa37/Beijing/2021 | F1.3P/ F31.5p |  |
| 28 | Fa38/Beijing/2021 | F1.3P/ F38.5p |  |
| 29 | Fa40/Beijing/2021 | F1.3P/ F17.5p |  |

**Supplementary Table 3** Pigeon circovirus (PiCV) references sequences obtained from GenBank and used in this study.

| Strain name | Year | Country | Host | Genome length (nt) | Accession number  (Genome sequence) | Accession number  (*cap* gene sequence) | Accession number  (*rep* gene sequence) |
| --- | --- | --- | --- | --- | --- | --- | --- |
| PiCV/Belgium/98-324/1998(*rep*) | 1998 | Belgium | pigeon | 954 |  |  | JX901125 |
| PiCV/Belgium/98-324/1998(*cap*) | 1998 | Belgium | pigeon | 822 |  | JX901125 |  |
| CoCV | unknown | Germany | racing pigeon | 2037 | AF252610 |  |  |
| CoCV | unknown | Germany | racing pigeon | 2037 | NC002361 |  |  |
| PL13 | 2002 | Poland | feral pigeon | 2041 | KF738846 |  |  |
| PL14 | 2002 | Poland | feral pigeon | 2034 | KF738847 |  |  |
| PL40 | 2002 | Poland | feral pigeon | 2042 | KF738855 |  |  |
| PL43 | 2002 | Poland | feral pigeon | 2038 | KF738856 |  |  |
| PL44B | 2002 | Poland | feral pigeon | 2043 | KF738858 |  |  |
| PL58 | 2002 | Poland | feral pigeon | 2038 | KF738862 |  |  |
| PL60 | 2002 | Poland | feral pigeon | 2038 | KF738863 |  |  |
| PL62 | 2002 | Poland | feral pigeon | 2038 | KF738864 |  |  |
| PL63 | 2002 | Poland | feral pigeon | 2041 | KF738865 |  |  |
| PL66B | 2002 | Poland | feral pigeon | 2034 | KF738867 |  |  |
| SRK/US/01 | 2003 | America | feral pigeon | 2041 | EU840176 |  |  |
| PL7 | 2003 | Poland | carrier pigeon | 2037 | KF738869 |  |  |
| PL44A | 2003 | Poland | carrier pigeon | 2038 | KF738857 |  |  |
| fj1 | 2009 | Fujian/China | carrier pigeon | 2037 | JN183455 |  |  |
| P98/01 | 2009 | Taiwan/China | pigeon | 822 |  | GQ844278 |  |
| PiCV1 | 2010 | Hungary | racing pigeon | 813 |  | JF330089 |  |
| PiCV2 | 2010 | Hungary | racing pigeon | 813 |  | JF330090 |  |
| PiCV3 | 2010 | Hungary | racing pigeon | 822 |  | JF330091 |  |
| PiCV4 | 2010 | Hungary | racing pigeon | 822 |  | JF330092 |  |
| PiCV5 | 2010 | Hungary | racing pigeon | 822 |  | JF330093 |  |
| PiCV6 | 2010 | Hungary | racing pigeon | 822 |  | JF330094 |  |
| PiCV7 | 2010 | Hungary | racing pigeon | 822 |  | JF330095 |  |
| PiCV9 | 2010 | Hungary | racing pigeon | 813 |  | JF330096 |  |
| PiCV14 | 2010 | Hungary | racing pigeon | 822 |  | JF330097 |  |
| PiCV15 | 2010 | Hungary | racing pigeon | 813 |  | JF330098 |  |
| PiCV/Japan/2/2010 | 2010 | Japan | racing pigeon | 2041 | LC035390 |  |  |
| PiCV/Belgium/11-07574/2011(*cap*) | 2011 | Belgium | pigeon | 813 |  | JX901127 |  |
| PL48 | 2011 | Poland | carrier pigeon | 2037 | KF738859 |  |  |
| PL53 | 2011 | Poland | carrier pigeon | 2035 | KF738860 |  |  |
| PL57 | 2011 | Poland | fancy pigeon | 2037 | KF738861 |  |  |
| PL66A | 2011 | Poland | fancy pigeon | 2037 | KF738866 |  |  |
| PL67 | 2011 | Poland | fancy pigeon | 2037 | KF738868 |  |  |
| PL89 | 2011 | Poland | fancy pigeon | 2037 | KF738870 |  |  |
| PL89X | 2011 | Poland | fancy pigeon | 2037 | KF738871 |  |  |
| PL94 | 2011 | Poland | carrier pigeon | 2037 | KF738872 |  |  |
| PL102 | 2011 | Poland | fancy pigeon | 2040 | KF738843 |  |  |
| PL114 | 2011 | Poland | fancy pigeon | 2038 | KF738844 |  |  |
| PL124 | 2011 | Poland | fancy pigeon | 2037 | KF738845 |  |  |
| PL170 | 2012 | Poland | farrier pigeon | 2036 | KF738848 |  |  |
| PL172 | 2012 | Poland | carrier pigeon | 2041 | KF738849 |  |  |
| PL177 | 2012 | Poland | carrier pigeon | 2037 | KF738850 |  |  |
| PL188 | 2012 | Poland | fancy pigeon | 2036 | KF738851 |  |  |
| PL189 | 2012 | Poland | fancy pigeon | 2036 | KF738852 |  |  |
| PiCV/P02/AUS | 2013 | Australia | feral pigeon | 2037 | MF136680 |  |  |
| PiCV/P03/AUS | 2013 | Australia | feral pigeon | 2039 | MF136681 |  |  |
| PiCV/P05/AUS | 2013 | Australia | feral pigeon | 2037 | MF136682 |  |  |
| PiCV/P08/AUS | 2013 | Australia | feral pigeon | 2037 | MF136684 |  |  |
| PiCV/P10/AUS | 2013 | Australia | feral pigeon | 2037 | MF136686 |  |  |
| PiCV/P11/AUS | 2013 | Australia | feral pigeon | 2037 | MF136687 |  |  |
| PiCV/P12/AUS | 2013 | Australia | feral pigeon | 2034 | MF136688 |  |  |
| PiCV/P13/AUS | 2013 | Australia | feral pigeon | 2039 | MF136689 |  |  |
| PiCV/P14/AUS | 2013 | Australia | feral pigeon | 2033 | MF136690 |  |  |
| PiCV/P15/AUS | 2013 | Australia | feral pigeon | 2037 | MF136691 |  |  |
| PiCV/P17/AUS | 2013 | Australia | feral pigeon | 2037 | MF136692 |  |  |
| AHBZ (*rep*) | 2013 | China | meat pigeon | 954 |  |  | KJ704801 |
| HBLF-E2 (*rep*) | 2013 | China | meat pigeon | 954 |  |  | KJ704802 |
| JSNJ (*rep*) | 2013 | China | meat pigeon | 954 |  |  | KJ704803 |
| NJPK (*rep*) | 2013 | China | meat pigeon | 954 |  |  | KJ704804 |
| SDDZ (*rep*) | 2013 | China | meat pigeon | 954 |  |  | KJ704805 |
| SHWH (*rep*) | 2013 | China | meat pigeon | 954 |  |  | KJ704806 |
| PL197 | 2013 | Poland | fancy pigeon | 2036 | KF738853 |  |  |
| PL201 | 2013 | Poland | fancy pigeon | 2043 | KF738854 |  |  |
| AF100 | 2014 | Anhui/China | meat pigeon | 2037 | KX108819 |  |  |
| AF104 | 2014 | Anhui/China | meat pigeon | 2037 | KX108824 |  |  |
| PR1625 | 2014 | Brazil | domestic pigeon | 2041 | KX808543 |  |  |
| RS0120 | 2014 | Brazil | domestic pigeon | 2041 | KY114965 |  |  |
| GF82 | 2014 | Guangdong/China | meat pigeon | 2031 | KX108805 |  |  |
| GF42 | 2014 | Guangdong/China | meat pigeon | 2037 | KX108780 |  |  |
| GF86 | 2014 | Guangdong/China | meat pigeon | 2034 | KX108781 |  |  |
| GF67 | 2014 | Guangdong/China | meat pigeon | 2040 | KX108783 |  |  |
| GF68 | 2014 | Guangdong/China | meat pigeon | 2037 | KX108785 |  |  |
| G2798 | 2014 | Guangdong/China | meat pigeon | 2037 | KX108786 |  |  |
| GF69 | 2014 | Guangdong/China | meat pigeon | 2037 | KX108787 |  |  |
| GH1811 | 2014 | Guangdong/China | meat pigeon | 2037 | KX108788 |  |  |
| GF85 | 2014 | Guangdong/China | meat pigeon | 2034 | KX108790 |  |  |
| GF103 | 2014 | Guangdong/China | meat pigeon | 2037 | KX108792 |  |  |
| GF54 | 2014 | Guangdong/China | meat pigeon | 2031 | KX108798 |  |  |
| GF71 | 2014 | Guangdong/China | meat pigeon | 2031 | KX108799 |  |  |
| GF104 | 2014 | Guangdong/China | meat pigeon | 2037 | KX108800 |  |  |
| GF81 | 2014 | Guangdong/China | meat pigeon | 2037 | KX108802 |  |  |
| GF46 | 2014 | Guangdong/China | meat pigeon | 2037 | KX108804 |  |  |
| GF17 | 2014 | Guangdong/China | meat pigeon | 2031 | KX108806 |  |  |
| GF53 | 2014 | Guangdong/China | meat pigeon | 2031 | KX108807 |  |  |
| GF13 | 2014 | Guangdong/China | meat pigeon | 2031 | KX108811 |  |  |
| GF43 | 2014 | Guangdong/China | meat pigeon | 2034 | KX108812 |  |  |
| GF87 | 2014 | Guangdong/China | meat pigeon | 2034 | KX108813 |  |  |
| GF84 | 2014 | Guangdong/China | meat pigeon | 2037 | KX108815 |  |  |
| GF80 | 2014 | Guangdong/China | meat pigeon | 2040 | KX108816 |  |  |
| GF16 | 2014 | Guangdong/China | meat pigeon | 2031 | KX108817 |  |  |
| GF90 | 2014 | Guangdong/China | meat pigeon | 2034 | KX108818 |  |  |
| GF88 | 2014 | Guangdong/China | meat pigeon | 2034 | KX108821 |  |  |
| GF45 | 2014 | Guangdong/China | meat pigeon | 2031 | KX108823 |  |  |
| GH1834 | 2014 | Guangdong/China | meat pigeon | 2031 | KX108825 |  |  |
| GF67 | 2014 | Jiangsu/China | meat pigeon | 2037 | KX108784 |  |  |
| JF9 | 2014 | Jiangsu/China | meat pigeon | 2040 | KX108793 |  |  |
| JF2 | 2014 | Jiangsu/China | meat pigeon | 2037 | KX108795 |  |  |
| JF3 | 2014 | Jiangsu/China | meat pigeon | 2040 | KX108797 |  |  |
| JF45 | 2014 | Jiangsu/China | meat pigeon | 2037 | KX108801 |  |  |
| JF8 | 2014 | Jiangsu/China | meat pigeon | 2031 | KX108809 |  |  |
| JF007 | 2014 | Jiangsu/China | meat pigeon | 2031 | KX108826 |  |  |
| GF64 | 2014 | Shanghai/China | meat pigeon | 2037 | KX108789 |  |  |
| SF76 | 2014 | Shanghai/China | meat pigeon | 2037 | KX108791 |  |  |
| SF85 | 2014 | Shanghai/China | meat pigeon | 2037 | KX108794 |  |  |
| SF78 | 2014 | Shanghai/China | meat pigeon | 2031 | KX108796 |  |  |
| SF80 | 2014 | Shanghai/China | meat pigeon | 2037 | KX108803 |  |  |
| SF81 | 2014 | Shanghai/China | meat pigeon | 2037 | KX108808 |  |  |
| SF335 | 2014 | Shanghai/China | meat pigeon | 2037 | KX108810 |  |  |
| SF86 | 2014 | Shanghai/China | meat pigeon | 2037 | KX108814 |  |  |
| SF82 | 2014 | Shanghai/China | meat pigeon | 2038 | KX108820 |  |  |
| SF83 | 2014 | Shanghai/China | meat pigeon | 2037 | KX108822 |  |  |
| SF079 | 2014 | Shanghai/China | meat pigeon | 2034 | KX108827 |  |  |
| SF77 | 2014 | Shanghai/China | meat pigeon | 2037 | KX108782 |  |  |
| JS15-1 | 2015 | China | racing pigeon | 2034 | KX431143 |  |  |
| SL-MA4/2016 | 2016 | Brazil | domestic pigeon | 2040 | MF664482 |  |  |
| SC-MA21/2016 | 2016 | Brazil | domestic pigeon | 2041 | MF664483 |  |  |
| Haikou | 2016 | Haikou/China | pigeon | 2038 | MG518478 |  |  |
| GC-MA1/2016 | 2016 | Brazil | domestic pigeon | 2038 | MF621931 |  |  |
| US 93A | Unknown | America | ornamental pigeon | 2037 | DQ915961 |  |  |
| US 002180 | Unknown | America | feral pigeon | 2036 | DQ915962 |  |  |
| Dove | Unknown | Australia | Senegal dove | 2039 | DQ915959 |  |  |
| Bel 18 | Unknown | Belgium | racing pigeon | 2032 | DQ915957 |  |  |
| Bel 20 | Unknown | Belgium | racing pigeon | 2032 | DQ915958 |  |  |
| Bel 936 | Unknown | Belgium | racing pigeon | 2038 | DQ915956 |  |  |
| Fra A40042 | Unknown | France | meat pigeon | 2037 | DQ915960 |  |  |
| Ita 4B | Unknown | Italy | meat pigeon | 2040 | DQ915950 |  |  |
| 7050 | Unknown | United Kingdom | racing pigeon | 2036 | AJ298230 |  |  |
| 9030 | Unknown | United Kingdom | racing pigeon | 2037 | AJ298229 |  |  |
| zj1 | Unknown | Zhejiang/China | meat pigeon | 2039 | DQ090945 |  |  |
| zj2 | Unknown | Zhejiang/China | meat pigeon | 2039 | DQ090944 |  |  |
| TF5/SN/2016 | 2016.11 | Shaanxi/China | racing pigeon | 822 |  | MW181901 |  |
| TY4/SN/2016 | 2016.12 | Shaanxi/China | racing pigeon | 816 |  | MW181902 |  |
| SX2/SN/2017 | 2017.05 | Shaanxi/China | racing pigeon | 816 |  | MW181903 |  |
| LT6/SN/2018 | 2018.03 | Shaanxi/China | racing pigeon | 954 |  |  | MW181904 |
| WQ7/SN/2018 | 2018.03 | Shaanxi/China | racing pigeon | 822 |  | MW181905 |  |
| WQ8/SN/2018 | 2018.03 | Shaanxi/China | racing pigeon | 819 |  | MW181906 |  |
| WQ9/SN/2018 | 2018.03 | Shaanxi/China | racing pigeon | 822 |  | MW181907 |  |
| WQ10/SN/2018 | 2018.03 | Shaanxi/China | racing pigeon | 822 |  | MW181908 |  |
| QYQX1/HE/2018 | 2018.04 | Hebei/China | racing pigeon | 822 |  | MW181909 |  |
| QYQX2/HE/2018 | 2018.04 | Hebei/China | racing pigeon | 822 |  | MW181910 |  |
| QYQX3/HE/2018 | 2018.04 | Hebei/China | racing pigeon | 822 |  | MW181911 |  |
| QYQX4/HE/2018 | 2018.04 | Hebei/China | racing pigeon | 813 |  | MW181912 |  |
| WL8/SN/2018 | 2018.05 | Shaanxi/China | racing pigeon | 822 |  | MW181913 |  |
| WL9/SN/2018 | 2018.05 | Shaanxi/China | racing pigeon | 822 |  | MW181914 |  |
| WL10/SN/2018 | 2018.05 | Shaanxi/China | racing pigeon | 825 |  | MW181915 |  |
| WL11/SN/2018 | 2018.05 | Shaanxi/China | racing pigeon | 825 |  | MW181916 |  |
| HD1/SN/2018 | 2018.07 | Shaanxi/China | racing pigeon | 813 |  | MW181917 |  |
| LH1/HE/2018 | 2018.08 | Hebei/China | racing pigeon | 816 |  | MW181918 |  |
| HP5/SN/2018 | 2018.09 | Shaanxi/China | racing pigeon | 822 |  | MW181919 |  |
| LQ4/SN/2018 | 2018.12 | Shaanxi/China | racing pigeon | 822 |  | MW181920 |  |
| JZ4/SN/2019 | 2019.02 | Shaanxi/China | racing pigeon | 822 |  | MW181921 |  |
| JZ5/SN/2019 | 2019.02 | Shaanxi/China | racing pigeon | 816 |  | MW181922 |  |
| JZ6/SN/2019 | 2019.02 | Shaanxi/China | racing pigeon | 816 |  | MW181923 |  |
| JZ7/SN/2019 | 2019.02 | Shaanxi/China | racing pigeon | 822 |  | MW181924 |  |
| TF1/SN/2016 | 2016.11 | Shaanxi/China | racing pigeon | 2037 | MW181925 |  |  |
| TF2/SN/2016 | 2016.11 | Shaanxi/China | racing pigeon | 2032 | MW181926 |  |  |
| TF3/SN/2016 | 2016.11 | Shaanxi/China | racing pigeon | 2032 | MW181927 |  |  |
| TF4/SN/2016 | 2016.11 | Shaanxi/China | racing pigeon | 2035 | MW181928 |  |  |
| TY1/SN/2016 | 2016.12 | Shaanxi/China | racing pigeon | 2037 | MW181929 |  |  |
| TY2/SN/2016 | 2016.12 | Shaanxi/China | racing pigeon | 2037 | MW181930 |  |  |
| TY3/SN/2016 | 2016.12 | Shaanxi/China | racing pigeon | 2044 | MW181931 |  |  |
| SX1/SN/2017 | 2017.05 | Shaanxi/China | racing pigeon | 2034 | MW181932 |  |  |
| YT1/SN/2017 | 2017.06 | Shaanxi/China | racing pigeon | 2042 | MW181933 |  |  |
| YT2/SN/2017 | 2017.06 | Shaanxi/China | racing pigeon | 2037 | MW181934 |  |  |
| YT3/SN/2017 | 2017.06 | Shaanxi/China | racing pigeon | 2037 | MW181935 |  |  |
| YT4/SN/2017 | 2017.06 | Shaanxi/China | racing pigeon | 2042 | MW181936 |  |  |
| YT5/SN/2017 | 2017.06 | Shaanxi/China | racing pigeon | 2042 | MW181937 |  |  |
| LT1/SN/2018 | 2018.03 | Shaanxi/China | racing pigeon | 2040 | MW181938 |  |  |
| LT2/SN/2018 | 2018.03 | Shaanxi/China | racing pigeon | 2039 | MW181939 |  |  |
| LT3/SN/2018 | 2018.03 | Shaanxi/China | racing pigeon | 2039 | MW181940 |  |  |
| LT4/SN/2018 | 2018.03 | Shaanxi/China | racing pigeon | 2042 | MW181941 |  |  |
| LT5/SN/2018 | 2018.03 | Shaanxi/China | racing pigeon | 2042 | MW181942 |  |  |
| WQ1/SN/2018 | 2018.03 | Shaanxi/China | racing pigeon | 2042 | MW181943 |  |  |
| WQ2/SN/2018 | 2018.03 | Shaanxi/China | racing pigeon | 2042 | MW181944 |  |  |
| WQ3/SN/2018 | 2018.03 | Shaanxi/China | racing pigeon | 2037 | MW181945 |  |  |
| WQ4/SN/2018 | 2018.03 | Shaanxi/China | racing pigeon | 2037 | MW181946 |  |  |
| WQ5/SN/2018 | 2018.03 | Shaanxi/China | racing pigeon | 2037 | MW181947 |  |  |
| WQ6/SN/2018 | 2018.03 | Shaanxi/China | racing pigeon | 2039 | MW181948 |  |  |
| KW1/SN/2018 | 2018.04 | Shaanxi/China | racing pigeon | 2032 | MW181949 |  |  |
| KW2/SN/2018 | 2018.04 | Shaanxi/China | racing pigeon | 2032 | MW181950 |  |  |
| KW3/SN/2018 | 2018.04 | Shaanxi/China | racing pigeon | 2032 | MW181951 |  |  |
| CA1/SN/2018 | 2018.04 | Shaanxi/China | racing pigeon | 2035 | MW181952 |  |  |
| CA2/SN/2018 | 2018.04 | Shaanxi/China | racing pigeon | 2041 | MW181953 |  |  |
| CA3/SN/2018 | 2018.04 | Shaanxi/China | racing pigeon | 2037 | MW181954 |  |  |
| CA4/SN/2018 | 2018.04 | Shaanxi/China | racing pigeon | 2035 | MW181955 |  |  |
| WL1/SN/2018 | 2018.05 | Shaanxi/China | racing pigeon | 2042 | MW181956 |  |  |
| WL2/SN/2018 | 2018.05 | Shaanxi/China | racing pigeon | 2042 | MW181957 |  |  |
| WL3/SN/2018 | 2018.05 | Shaanxi/China | racing pigeon | 2042 | MW181958 |  |  |
| WL4/SN/2018 | 2018.05 | Shaanxi/China | racing pigeon | 2044 | MW181959 |  |  |
| WL5/SN/2018 | 2018.05 | Shaanxi/China | racing pigeon | 2037 | MW181960 |  |  |
| WL6/SN/2018 | 2018.05 | Shaanxi/China | racing pigeon | 2037 | MW181961 |  |  |
| WL7/SN/2018 | 2018.05 | Shaanxi/China | racing pigeon | 2037 | MW181962 |  |  |
| QD1/SN/2018 | 2018.07 | Shaanxi/China | racing pigeon | 2032 | MW181963 |  |  |
| QD2/SN/2018 | 2018.07 | Shaanxi/China | racing pigeon | 2032 | MW181964 |  |  |
| QD3/SN/2018 | 2018.07 | Shaanxi/China | racing pigeon | 2042 | MW181965 |  |  |
| QD4/SN/2018 | 2018.07 | Shaanxi/China | racing pigeon | 2037 | MW181966 |  |  |
| QD5/SN/2018 | 2018.07 | Shaanxi/China | racing pigeon | 2037 | MW181967 |  |  |
| QD6/SN/2018 | 2018.07 | Shaanxi/China | racing pigeon | 2035 | MW181968 |  |  |
| BYHL1/BJ/2018 | 2018.08 | Beijing/China | racing pigeon | 2032 | MW181969 |  |  |
| DS1/GS/2018 | 2018.08 | Gansu/China | racing pigeon | 2037 | MW181970 |  |  |
| DFSM1/QH/2018 | 2018.08 | Qinghai/China | racing pigeon | 2041 | MW181971 |  |  |
| DFSM2/QH/2018 | 2018.08 | Qinghai/China | racing pigeon | 2041 | MW181972 |  |  |
| JZ1/SN/2018 | 2018.08 | Shaanxi/China | racing pigeon | 2037 | MW181973 |  |  |
| HP1/SN/2018 | 2018.09 | Shaanxi/China | racing pigeon | 2041 | MW181974 |  |  |
| HP2/SN/2018 | 2018.09 | Shaanxi/China | racing pigeon | 2037 | MW181975 |  |  |
| HP3/SN/2018 | 2018.09 | Shaanxi/China | racing pigeon | 2032 | MW181976 |  |  |
| HP4/SN/2018 | 2018.09 | Shaanxi/China | racing pigeon | 2035 | MW181977 |  |  |
| YB1/SN/2018 | 2018.10 | Shaanxi/China | racing pigeon | 2042 | MW181978 |  |  |
| YB2/SN/2018 | 2018.10 | Shaanxi/China | racing pigeon | 2037 | MW181979 |  |  |
| YB3/SN/2018 | 2018.10 | Shaanxi/China | racing pigeon | 2037 | MW181980 |  |  |
| YB4/SN/2018 | 2018.10 | Shaanxi/China | racing pigeon | 2034 | MW181981 |  |  |
| DA1/XJ/2018 | 2018.10 | Xinjiang/China | racing pigeon | 2030 | MW181982 |  |  |
| LQ1/SN/2018 | 2018.12 | Shaanxi/China | racing pigeon | 2035 | MW181983 |  |  |
| LQ2/SN/2018 | 2018.12 | Shaanxi/China | racing pigeon | 2041 | MW181984 |  |  |
| LQ3/SN/2018 | 2018.12 | Shaanxi/China | racing pigeon | 2045 | MW181985 |  |  |
| JZ2/SN/2019 | 2019.02 | Shaanxi/China | racing pigeon | 2035 | MW181986 |  |  |
| JZ3/SN/2019 | 2019.02 | Shaanxi/China | racing pigeon | 2041 | MW181987 |  |  |
| LH2/HE/2019 | 2019.04 | Hebei/China | racing pigeon | 2037 | MW181988 |  |  |
| LH3/HE/2019 | 2019.04 | Hebei/China | racing pigeon | 2042 | MW181989 |  |  |
| QD7/SN/2019 | 2019.04 | Shaanxi/China | racing pigeon | 2037 | MW181990 |  |  |
| QD8/SN/2019 | 2019.08 | Shaanxi/China | racing pigeon | 2035 | MW181991 |  |  |

**Supplementary Table 4** Details of recombination events (GenBank accession numbers) detected in the Pigeon circovirus (PiCV) strains obtained in this study.

| **Event** | **Genome position** | **Recombinant** | **Potential minor parent(s)** | **Potential major parent(s)** | **Detection methods** | ***p-value*** |
| --- | --- | --- | --- | --- | --- | --- |
| 1 | 2020-1138 | **OR843262**  **OR843257**  **OR843258**  **OR843259**  **OR843260**  **OR843261**  KF738855  DQ090944 | **ON598385** | MW181941 （Shaanxi/China  ） | RGBMCS**T** | 6.88×10^-27^ |
| 2 | 2012-1129 | **ON598386** | MW181938 (Shaanxi/China) | KX108795 (Jiangsu/China) | RBMCS**T** | 2.50×10^-22^ |
| 3 | 821-1995 | **OR843274**^‡^  KF738849 | DQ090945 (Zhejiang/China) | MW181972 (Qinghai/China) | RGBMC**S**T | 2.24×10^-32^ |
| 4 | 743-2021 | **OR843272**^‡^ | MF664482 (Brazil) | **OR843277** | RGBMC**S**T | 5.14×10^-29^ |
| 5 | 722-1927 | **OR843271**^‡^ | KF738870 Poland | KY114965 Brazil | RGBMS**T** | 4.02×10^-20^ |
| 6 | 312-759 | **ON598388**^‡^ | unknown | **ON598384** | **R**GBMCST | 5.49×10^-13^ |
| 7 | 175-1139 | **OR843266**  MW181986  MW181989  **OR843274** | KX108784(Jiangsu/China) | MW181953 Shaanxi/China | RMC**S**T | 1.36×10^-7^ |
| 8 | 133-721^†^ | **OR843271** | KX108824(Anhui/China) | KF738865(Poland) | MC**T** | 2.47×10^-6^ |
| 9 | 158^†^-1140 | **OR843276**^‡^  **OR843277**  MW181952  MW181977  MW181963 | Unknown | MW181943 (Shaanxi/China) | **M**CT | 1.58×10^-5^ |
| 10 | 1155-1353^†^ | ON598384^‡§^  ON598387  ON598388  MW181965 | MF136692(Australia) | **OR843273** | M**S**T | 1.89×10^-3^ |
| 11 | 318-528 | **OR843255**  **OR843256**  MW181969  MW181927  MW181981  MW181932  MW181976  MW181955  DQ915957 | MW181975(Shaanxi/China) | MW181968(Shaanxi/China) | **R**CT | 1.90×10^-3^ |
| 12 | 1342-1886^†^ | **OR843257**^‡^  **OR843258**  **OR843259**  **OR843260**  **OR843261**  **OR843262**  **OR843266**  **OR843276**  MW181938  MW181933  MW181936  MW181937  MW181989  MW181943  MW181953  MW181956  MW181957  MW181958  MW181974  MW181984  MW181987  KF738855  KF738858  MF664482  DQ090944 | Unknown | MW181986(Shaanxi/China) | RGMC**S**T | 6.34×10^-10^ |
| 13 | 1355-1939 | AF252610^‡^  NC_002361  DQ915960  KF738868  KF738871  KF738851  KF738861  KF738852  KF738853  KX108801  MW181929  MW181925  KX108822  MW181970  MW181965  KF738859  KF738866  KF738870  AJ298229  AJ298230  MF136680  MF136682  MF136684  MF136686  MF136687  MF136691  MF136692  MF136645  KF738872  KF738848  KX108819  KX108795  KX108789  **ON598386**  **ON598384**  **ON598388**  **ON598387**  KF738845  DQ915961  DQ915962  MW181988  MW181990  KX108824  KX108820  KX108808  KX108786  KX108802  KX108803  KX108792  KX108782  KX108814  KX108815  KX108794  KX108799  KX108823  KX108804  KF738860 | MW181968(Shaanxi/China) | MW181973(Shaanxi/China) | MC**S**T | 1.40×10^-8^ |

***Note.*** Methods used to detect recombination are RDP (R), GENCONV (G), Bootscan (B), MaxChi (M), Chimaera (C), Siscan (S) and 3Seq (T). The method with the most significant associated *p*-value is indicated in bold for each event. The GenBank accession numbers related to PiCV isolates in this study are highlighted in bold font.

^†^The actual breakpoint position is undetermined.

^‡^The recombinant sequence may have been misidentified.

^§^The recombination signal could have been caused by an evolutionary process other than recombination.
